# Incomplete reverse remodeling in pulmonary hypertension‐induced right ventricular dysfunction in aged mice

**DOI:** 10.14814/phy2.70422

**Published:** 2025-06-11

**Authors:** Benjamin D. McNair, Sushumna B. Satyanarayana, Julian M. Matthews, Sydney M. Polson, Emma R. Mehl, Joshua P. Thornburg, Danielle R. Bruns

**Affiliations:** ^1^ Kinesiology & Health University of Wyoming Laramie Wyoming USA

**Keywords:** aging, hypoxia, reverse remodeling, right ventricle

## Abstract

Right ventricular (RV) function is the strongest predictor of survival in pulmonary hypertension (PH) and age‐related heart disease; however, no therapies improve RV function. Understanding how the RV undoes pathological remodeling (reverse remodeling) might aid in identifying therapies, particularly in aging populations in which RV failure is significant. Our objective was to determine if the aged RV can undergo reverse remodeling following the resolution of pathological afterload by pulmonary hypertension (PH). We exposed male and female aged (18–21 months) C57BL/6 mice to hypobaric hypoxia (HH) for 4 weeks to model PH before returning the mice to normoxia for three (WK3RR) or six (WK6RR) weeks. HH stimulated RV hypertrophy and fibrosis which were attenuated with WK3RR and WK6RR. Activation of autophagy and anti‐fibrotic pathways likely underlie morphological reverse remodeling. However, HH decreased RV systolic function as assessed by fractional area change (FAC) and stroke volume (SV) that were not rescued with normoxia re‐exposure. The aged RV can undergo morphological reverse remodeling following the removal of pathological load; however, RV function does not improve. Further investigation into the mechanisms of reverse remodeling may identify potential drug therapies for maladaptive RV remodeling with aging.

## 1 INTRODUCTION

Pulmonary hypertension (PH) is a progressive and incurable condition that over time causes right ventricle (RV) remodeling and eventual RV failure (Montani et al., 2013). PH is becoming increasingly common and devastating in aging populations in whom the risk for PH and RV failure is high (Owan et al., 2006) and RV dysfunction is significant compared to younger cohorts (McNair et al., 2023). Advanced age is the single most powerful risk factor for heart disease (Mozaffarian, Benjamin et al., 2015), increasing risk by an order of magnitude more than traditional cardiovascular risk factors. Age‐associated changes in cardiac repair and stress responses render aged hearts at higher risk of worse outcomes compared to younger models (Dai & Rabinovitch, 2009) and suggest that understanding how the aged heart responds to stress will be necessary to treat PH and RV failure in aged populations.

Survival of patients with PH is ultimately determined by RV function (van de Veerdonk et al., 2011). Of the few therapeutic options presently available for PH, none directly target or improve RV function. Further, by the time treatment is initiated, typically in response to the presence of symptoms, changes in RV structure and function have already occurred. Therefore, there remains a need to identify therapies that undo RV remodeling or reverse remodel the RV. Current therapies for PH target the pulmonary vasculature through stimulation of vasorelaxation, thus lowering RV afterload. Presumably, the attenuation in afterload also results in regression of RV remodeling, but in many cases, this has not yet been experimentally tested or remains unclear. In a young rabbit model of pulmonary artery banding (PAB) where the PA is surgically constricted to cause PH and increased RV afterload, deflation of the PA band over 6 weeks resulted in mild regression of RV size and some improvements in RV systolic function (Fujioka et al., 2022). A longer recovery time of 12 weeks in young male mice post‐PAB demonstrated morphological and functional RV recovery with disintegration of the suture (Boehm et al., 2020). Clinically, there is modest evidence for RV reverse remodeling in young PH patients (mean age 33 years) that underwent single lung transplant. Three months post‐transplant, RV performance normalized to healthy control values, and RV mass normalized within a year (Frist et al., 1995). However, the extent of the reverse remodeling was highly variable, with the greatest improvement in RV function occurring in younger patients with shorter duration of disease (Schulman et al., 1996). Therefore, it remains unclear whether older populations maintain the ability to reverse remodel the RV. Given the growing number of aging patients diagnosed with PH and RV failure (Hoeper et al., 2016), addressing this need is significant. Accordingly, the purpose of the present study was to understand whether HH induced RV remodeling could be reversed in an aged model of hypoxia‐induced PH. We demonstrate that although the RV morphologically recovered with afterload resolution, RV performance did not improve, highlighting the need to understand how aging impacts RV function and the response to and resolution from stress.

## 2 METHODS

### 2.1 Mouse model of hypoxic pulmonary hypertension and RV dysfunction

Aged (18–21 months) C57BL6 male and female mice were donated from the National Institute of Aging. Aged mice acclimated at the University of Wyoming for 1 week before beginning the experimental paradigm. All protocols were approved by the University of Wyoming IACUC, and all animals were housed in the same vivarium. Mice were housed two to five animals per cage in a temperature‐controlled room (21°C) on a standard 12/12 light/dark cycle. Food (Lab Diet 5001) and water were provided ad libitum. Mice were placed in a hypobaric hypoxia (HH) chamber (5000 m) for 4 weeks (McNair et al., 2023). During HH exposure, mice were briefly removed from the chamber (<1 h) for cage changes and collection of weekly body weight before returning to HH. Following 4 weeks of HH, mice were returned to local ambient altitude for 1, 3, and 6 weeks. Preliminary findings showed that 1 week in normoxia was not long enough to induce reverse remodeling; thus, only 3‐ and 6‐week values are presented. Body weight, water weight, and food weight were measured weekly. The mice were on a normal 12 h light/dark cycle. Food was removed the night before, and the mice were sacrificed using pentobarbital injection. At sacrifice, the RV was dissected from the left ventricle (LV) and septum and flash‐frozen for biochemical analyses. Tibia length (TL) was measured by caliper to normalize RV masses to animal body size.

### 2.2 Echocardiography

Cardiac function was assessed via transthoracic echocardiography (Visual Sonics Vevo 2100; FujiFilm). Mice were induced with 2.5% isoflurane in compressed air and maintained between 1% and 3% to permit a heart rate >400 BPM. Core temperature was monitored and maintained at 37°C with a heating platform (FujiFilm). Respiratory rate and heart rate were monitored with 4 lead limb ECG (FujiFilm). Parasternal short axis views of the RV were captured in B‐Mode and M‐Mode. Parasternal long axis views of the RV and pulmonary artery (PA) were captured in B‐Mode. Pulse wave Doppler views of the PA were captured for PA acceleration times (PAT). RV M‐Mode tracings were used to assess ventricle wall thickness during systole and diastole (RVFW;s and RVFW;d). Short axis B‐Mode tracings were used to assess fractional area change (FAC). Pulse wave Doppler views were traced for PA acceleration time (PAT), a surrogate for mPAP (Yared et al., 2011). RV stroke volume (SV) and cardiac output (CO) were calculated by Visual Sonics software.

### 2.3 Immunoblotting

RV were homogenized in RIPA, and protein concentration was determined by BCA. Equal quantity of protein was separated by SDS‐PAGE electrophoresis and transferred to nitrocellulose membranes. Membranes were blocked in 5% bovine serum albumin before overnight incubation in primary antibody. Membranes were washed and incubated with secondary antibody. For phospho‐specific antibodies, membranes were stripped, visualized to verify removal of antibody, then incubated overnight with total antibody. Protein bands were visualized with chemiluminescent substrate (ThermoFisher #34578) on Analytik Jena imager and quantified in ImageJ. Primary antibodies included: ANP (Santa Cruz 515701), phospho‐Erk1/2 (Cell Signaling 4370), total Erk1/2 (Cell Signaling 4695), αSMA (Sigma A2547), actinin (Abcam 68167), LC3 A/B (Cell Signaling 4108). Secondary antibodies included (mouse Sigma A21140 and rabbit: Invitrogen A11008).

### 2.4 qRT‐PCR

RNA was extracted from the RV using standard Trizol protocols and reverse transcribed using High‐Capacity RNA‐to‐cDNA™ Kit (ThermoFisher #4387406). Real‐time RT‐PCR was performed using iQ SYBR Green Supermix (ThermoFisher #A46109) and normalized to the housekeeping gene β‐actin. Expression was calculated by ΔΔCt relative to Con within sex and expressed as fold change. Primer sequences were as follows: ANF Forward: GCCGGTAGAAGATGAGGTCATG, ANF Reverse: GCTTCCTCAGTCTGCTCACTCA; BNP Forward: CGCTGGGAGGTCACTCCTAT, BNP Reverse: GCTCTGGAGACTGGCTAGGACTT, SPARC Forward: CACCTGGACTACATCGGACCAT, SPARC Reverse: CTGCTTCTCAGTGAGGAGGTTG; MMP1 Forward: AGGAAGGCGATATTGTGCTCTCC, MMP1 Reverse: TGGCTGGAAAGTGTGAGCAAGC; myostatin Forward: TCACGCTACCACGGAAACAA; myostatin Reverse: AGGAGTCTTGACGGGTCTGA; Bcl2 Forward: CACCCCTGGTGGACAACATC; Bcl2 Reverse: ATAGTTCCACAAAGGCATCCCAG; Bax Forward: TGCTAGCAAACTGGTGCTCA; Bax Reverse: AGTAGGAGAGGAGGCCCAGC; 18s Forward: GCCGCTAGAGGTGAAATTCTTG, 18s Reverse: CTTTCGCTCTGGTCCGTCTT.

### 2.5 Histology

RV were frozen in Optimal Cutting Temperature buffer (Fisher #4585) and 4 μm sections were cut using a pre‐cooled cryostat (Leica Biosystems). For RV myocyte cell area, sections were fixed with 3% paraformaldehyde, then permeabilized with 1% Triton X‐100. Slides were washed three times with deionized water and stained with wheat germ agglutinin‐488 (ThermoFisher W11261) in the dark. After incubation, samples were washed with PBS and mounted for imaging by fluorescent microscopy (Olympus IX71). Cardiomyocyte area was calculated using ImageJ in a blinded manner by tracing all clear and complete cells per picture. To quantify fibrosis, RV were stained following standard Picro‐sirius red (Abcam 246832) protocols. Images were acquired at 6X and processed with ImageJ. To quantify red‐stained collagen, the scale bar in ImageJ was altered to μm and converted to gray scale. Red‐stained collagen is segmented from other color contrasts using color thresholding. The red color threshold was adjusted using a Con and HH RV sample to determine estimated min and max. The same color threshold was used to quantify the amount of stained collagen for all groups and expressed as percent area. Each sex and condition had at least 3 biological replicates, and images were captured from at least 6 views.

### 2.6 Statistical analyses

Data were analyzed by two‐way ANOVA and Fisher's LSD post hoc testing for within‐group differences in the presence of a significant main effect and interaction. Significance was set a priori at *α* < 0.05. Statistical tests were performed using Prism. Data are expressed as mean ± standard deviation (SD).

## 3 RESULTS

**FIGURE 1:**
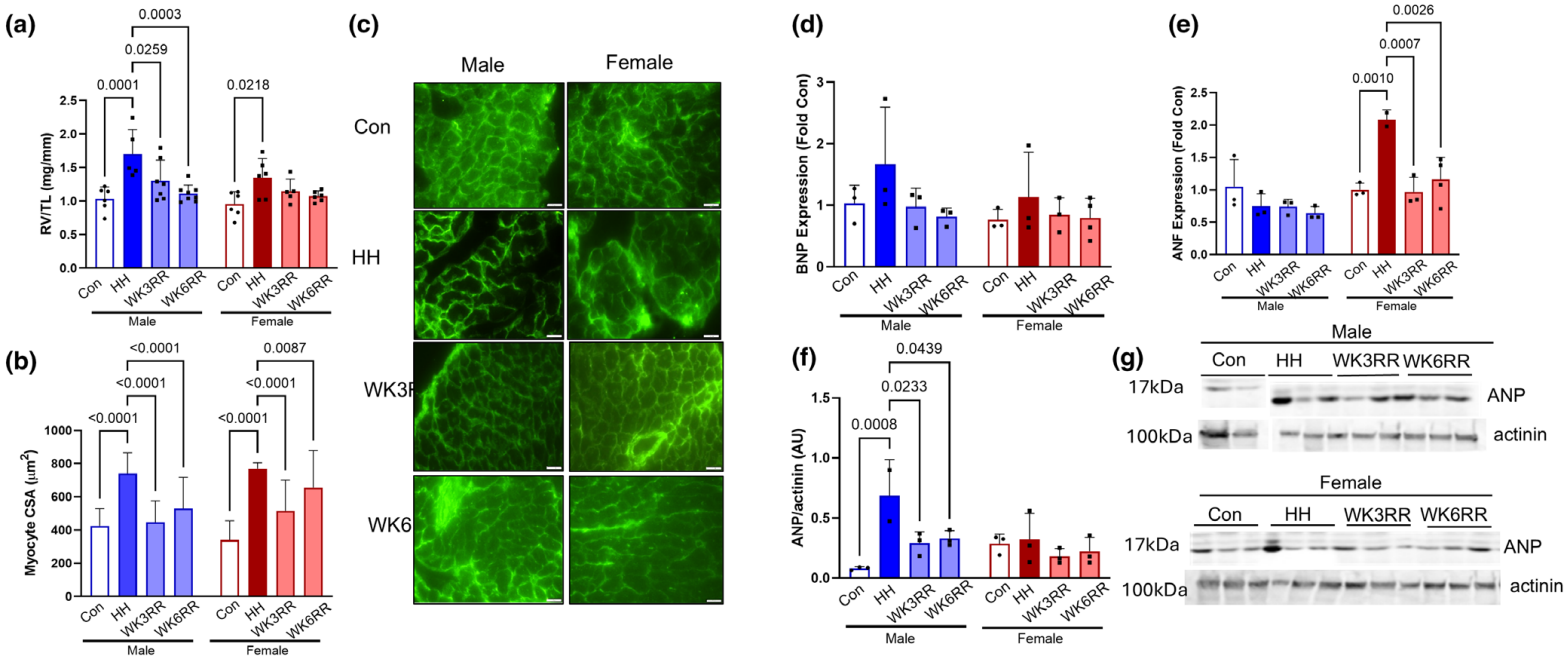
Hypertrophic remodeling in aged mice exposed to hypobaric hypoxia (HH) for 4 weeks then re‐exposed to normoxia for 3 and 6 weeks (WK3RR and WK6RR). (a) RV weight normalized to TL (RV/TL) and (b) RV cardiomyocyte cross sectional area (CSA) were higher in response to HH and reduced during normoxia exposure in both male and female mice. (c) Representative images of lectin staining. (d) BNP expression was upregulated in aged males and females during HH and decreased during WK3RR. (e) ANF expression increased in aged females in response to HH and returned to control by WK3RR. (f) Protein expression of ANP was upregulated in males and decreased during WK3RR. (g) Representative images. Data were assessed by two‐way ANOVA. Data are expressed as mean ± SD. For histology, *n* = 3 mice/group with at least 100 cells. Scale bar = 20 μm.

As expected, aged mice showed hypertrophic remodeling in response to HH as evidenced by elevated RV mass normalized by TL (RV/TL) and larger RV myocyte cross‐sectional area (CSA) (Figure 1). When mice returned to normoxic conditions, RV/TL and RV CSA regressed in size and area by WK3RR (Figure 1a–c). Expression of the pro‐hypertrophic gene B‐type natriuretic protein (BNP) was unchanged by HH or reverse remodeling; however, atrial natriuretic factor (ANF) expression increased with HH in females and reversed during WK3RR (Figure 1d,e). At the protein level, HH strongly induced ANP expression in the male RV that reversed with WK3RR and WK6RR (Figure 1f,g).

**FIGURE 2:**
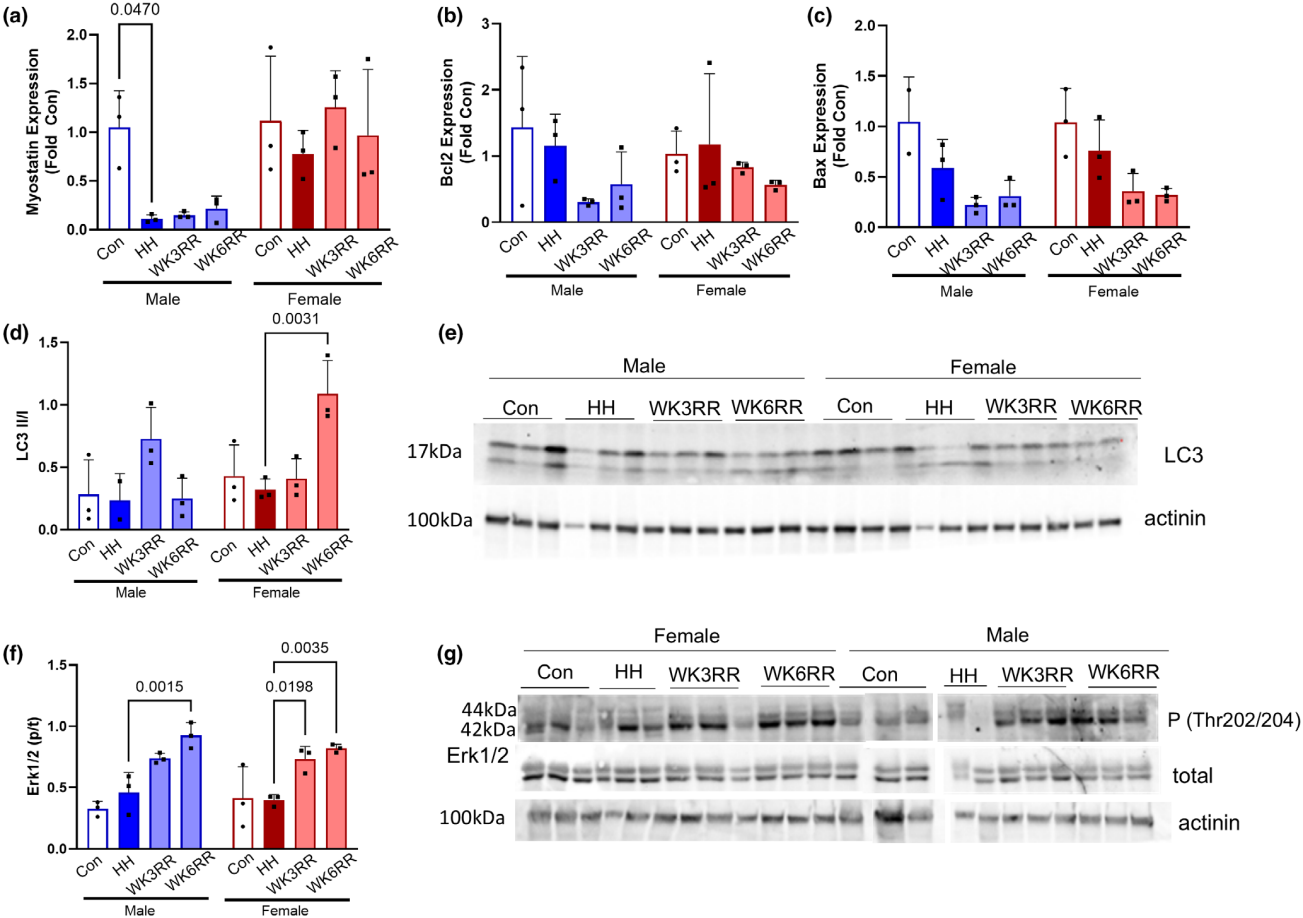
Mechanisms of atrophy in the RV in response to reverse remodeling (RR). (a) Myostatin expression was lower in the hypoxic (HH) male RV compared to control but did not normalize during normoxia. (b, c) Unchanged apoptosis regulators in the aged hypoxic and reverse remodeled RV. (d) Expression of autophagy regulator LC3 tended to increase with WK3RR male and reached significance at WK6RR in female mice. (e) Representative immunoblot. (f) Extracellular signal‐regulated kinase (Erk1/2) activation was higher at WK3RR and WK6RR compared to HH. (g) Representative immunoblot. Data were assessed by two‐way ANOVA. Data are expressed as mean ± SD.

Molecular mechanisms for regression of myocyte size are not fully clear, but studies of cardiac cachexia induced myocyte atrophy suggest apoptosis or autophagy may be responsible (Murphy, 2016). Consistent with RV hypertrophy, we found downregulation of myostatin expression in the male RV with HH that did not normalize with normoxia (Figure 2a). Expression of pro‐ and anti‐apoptotic regulators Bcl2 and Bax did not differ with HH or reverse remodeling (Figure 2b,c). Expression of the autophagy regulator was higher at WK6RR in the female, perhaps suggesting activation of autophagy as a mechanism of loss of RV mass in the female heart (Figure 2d,e). Extracellular signal‐regulated kinase (Erk1/2) signaling regulates apoptosis and autophagy (Cagnol & Chambard, 2010) and is involved in left ventricular (LV) reverse remodeling (Baba et al., 2003). Therefore, we quantified activation of Erk in the RV. Although Erk1/2 activation did not change with HH, upon return to normoxia, Erk1/2 activation was higher at WK3RR and WK6RR in both male and female mice compared to HH (Figure 2f,g).

**FIGURE 3:**
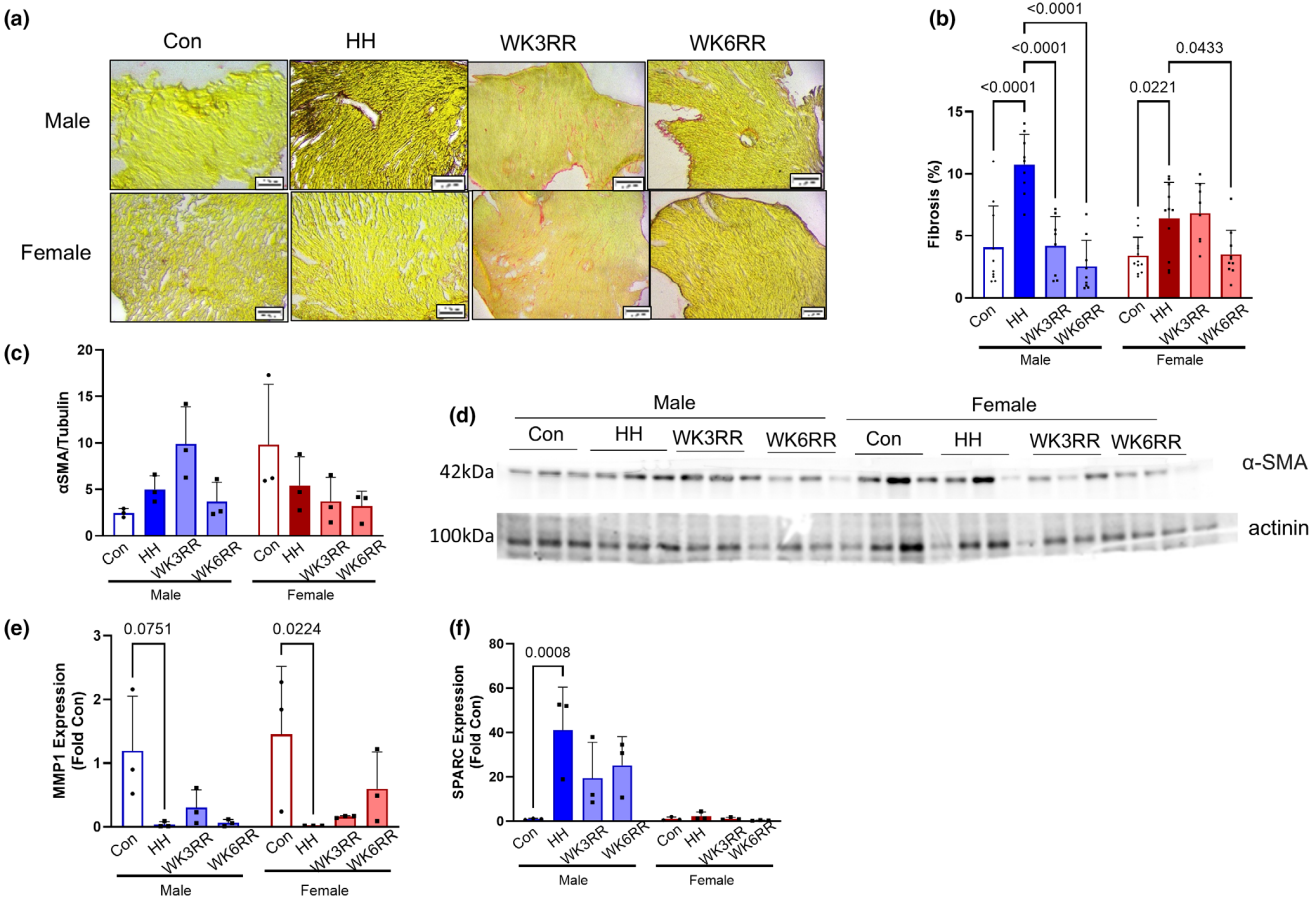
RV fibrosis in hypobaric hypoxia (HH) and reverse remodeling. (a) Representative Picrosirius red stains for collagen. (b) Quantification of collagen deposition as a percentage of area. HH induced collagen deposition in aged males and females that was reduced after return to normoxia. (b) α‐SMA expression was largely unchanged with HH and return to hypoxia. (d) Representative western blot images. (e) MMP‐1 expression was downregulated in both males and females with HH but did not normalize with normoxia. (f) SPARC was upregulated in males in response to HH and partially normalized at WK6RR. Expression was unchanged in females. For histology, *n* = 3 mice/group with at least 6 views. Scale bar = 20 μm. Data were assessed by two‐way ANOVA. Data are expressed as mean ± SD.

PH‐induced RV dysfunction is also accompanied by fibrotic remodeling (Al‐Qazazi et al., 2022) thus, we quantified collagen by histology. HH induced collagen deposition (Figure 3a). During normoxia re‐exposure, collagen deposition was attenuated by WK3RR in male and WK6RR in female mice (Figure 3a,b). α smooth muscle actin (αSMA) expression was unchanged with HH or return to normoxia (Figure 3c,d). Matrix metalloproteinase 1 (MMP1), known for breaking down collagen type 1, was downregulated in HH in both males and females and trended to increase with reverse remodeling (Figure 3e). Expression of secreted protein acidic and rich in cysteine (SPARC), a marker of collagen cross‐linking, was upregulated upon HH exposure in males and trended towards normalization with normoxia (Figure 3f).

**FIGURE 4:**
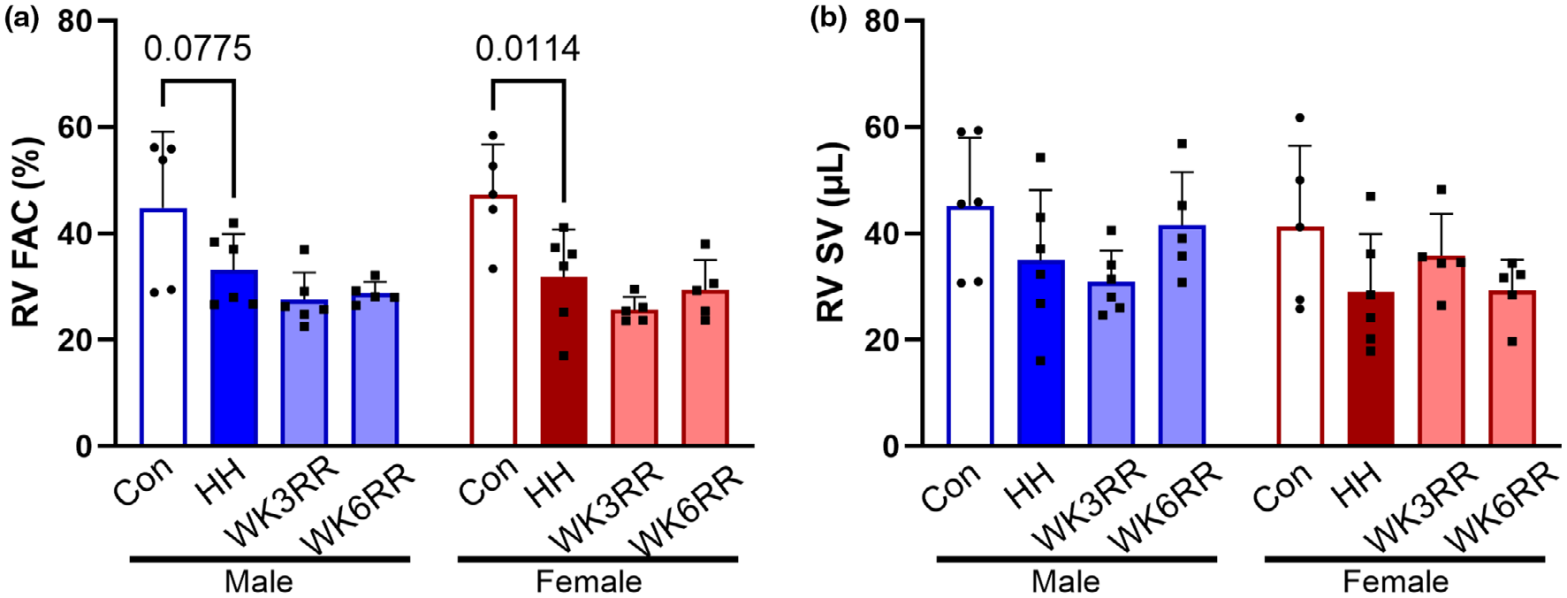
RV function by echocardiography in response to hypobaric hypoxia (HH) and reverse remodeling (RR) with return to normoxia. (a) RV fractional area change (FAC) was reduced in HH in males and females and did not recover in WK3RR or WK6RR. (b) RV stroke volume (SV), reduced upon HH exposure, and did not return to normal during WK3RR in males. In females, it slightly showed an increase, but neither reached to normal nor significance. Data were assessed by two‐way ANOVA. Data are expressed as mean ± SD.

To determine whether the reversal of hypertrophy and fibrosis was also accompanied by improved RV function, we performed echocardiography (Table 1). RV fractional area change (FAC) was reduced with HH but was unchanged by normoxia re‐exposure (Figure 4a). SV was reduced during HH and did not recover with return to normoxia (Figure 4b).

**TABLE 1:** Summary of echocardiography in aged mice in response to hypobaric hypoxia (HH) and return to normoxia for 3 (RRWK3) and 6 (RRWK6) weeks.

	Male				Female			
	Con	HH	WK3RR	WK6RR	Con	HH	WK3RR	WK6RR
HR (bpm)	454 ± 40	420 ± 21	514 ± 37	543 ± 29	479 ± 48	446 ± 9	496 ± 44	495 ± 98
PAT (ms)	16.6 ± 2.5	18.0 ± 1.3	21.6 ± 4.8	20.8 ± 1.4	18.6 ± 3.5	15.3 ± 1.7	17.5 ± 2.8	18.7 ± 3.9
RV wall;s (mm)	0.67 ± 0.22	0.58 ± 0.16	0.60 ± 0.14	0.64 ± 0.04	0.68 ± 0.17	0.73 ± 0.16	0.57 ± 0.11	0.60 ± 0.07
RV wall;d (mm)	0.41 ± 0.13	0.43 ± 0.1	0.37 ± 0.11	0.40 ± 0.04	0.41 ± 0.12	0.52 ± 0.1	0.34 ± 0.1*	0.33 ± 04*
RV Area;s (mm^2^)	3.9 ± 1.0	5.4 ± 1.6	6.1 ± 1.1	6.6 ± 1.1	3.5 ± 1.2	5.4 ± 0.2	7.0 ± 0.6	6.6 ± 0.9
RV Area;d (mm^2^)	6.8 ± 1.2	8.0 ± 2.2	7.7 ± 1.4	9.2 ± 1.3	6.8 ± 1.3	7.8 ± 0.7	9.3 ± 0.8	9.3 ± 0.8
FAC (%)	41 ± 16	33 ± 8*	28 ± 5	29 ± 5	47 ± 4	33 ± 9*	26 ± 2	29 ± 5
SV (μL)	45.3 ± 12.7	31.1 ± 11	30.8 ± 5.9	37.7 ± 6.0	41.3 ± 14.2	30.7 ± 11	35.8 ± 7.9	29.3 ± 2.0

*Note*: RV wall thickness during diastole (RV wall; mm) thinned by WK3 and WK6RR in female mice (*p = 0.02 and *p* = 0.01, respectively). RV area during systole and diastole (RV area; mm^2^) were larger in male and female mice with HH and did not normalize with normoxia. RV fractional area change (FAC) declined in both male and female mice with HH (*p = 0.05 and *p = 0.01, respectively) but did not improve with return to normoxia. Stroke volume (SV) also trended to decline with HH and was not improved with reverse remodeling. Data were assessed by two‐way ANOVA. Data are expressed as mean ± SD. *n =* 4–6 per group.

Although our primary objective was to determine if the aged RV can reverse remodel, we compared our findings to the young adult (4 month) that has been reported to undergo complete reversal of RV size and mass (Fujioka et al., 2022). Indeed, we did find that young mice also showed regression of myocyte CSA upon return to normoxia (Figure SA,C) and regression of collagen content (Figure SB). Young mice also showed reversal of RV FAC in adult female mice and improved RV SV in adult male mice with return to normoxia (Figure SD,E).

## 4 DISCUSSION

RV dysfunction predicts survival in age‐related cardiopulmonary conditions including PH (Goliasch et al., 2015; van de Veerdonk et al., 2011). Despite the importance of maintaining RV function on patient outcomes, no therapies exist that improve RV function. Many PH treatments decrease pulmonary pressure, relieving RV afterload in an attempt to improve RV function. However, the RV undergoes pathological remodeling in response to PH, and whether the RV can reverse remodel these pathological changes and improve function remains to be investigated in aged models. Thus, we sought to test whether the aged RV could reverse remodel after resolution of PH. We demonstrate that the aged RV, like the young RV, possesses mechanisms to reverse HH‐induced RV hypertrophy and fibrosis. However, despite reversal of hypertrophic and fibrotic remodeling, removal of pathological load does not rescue RV function, rendering the reverse remodel incomplete. Future efforts will clarify the mechanisms by which this reverse remodeling occurs to elucidate RV‐specific drug targets for the aging heart.

Exposure to HH induced hypertrophic RV growth that normalized upon return to normoxia. Identification of mechanisms of atrophy in the setting of reverse remodeling may aid in the development of targeted therapies. One such mechanism that has received attention in the model of LV reverse remodeling is the activity of the signaling kinase Erk, which dramatically decreases after mechanical unloading by LV assist devices (LVAD) (Baba et al., 2003). Similarly, genetic inhibition of Erk activation blocked PA smooth muscle cell proliferation, suggesting it may aid in blunting cardiopulmonary remodeling in the setting of PH (Ferguson et al., 2021). Neither of these previous reports, however, has measured Erk1/2 activation in the RV, nor in the context of aging. We found that Erk1/2 activation is higher with reverse remodeling in the aged RV compared to HH. Although context dependent, Erk is generally linked to anti‐apoptotic outcomes in cardiomyocytes (Zhang et al., 2015). Given the activation of Erk in the reverse remodeling RV, we were surprised to find that the expression of pro‐ and anti‐apoptotic regulators Bcl2 and Bax were unchanged with HH and with return to normoxia, though we do acknowledge we did not measure apoptosis directly. Erk also regulates autophagy, which is typically associated with classical markers of autophagy, such as induction of LC3 (Cagnol & Chambard, 2010). Consistent with increased activity of Erk1/2 during WK3RR and WK6RR, we also found activation of LC3 in the female RV, perhaps suggesting RV autophagy‐mediated mechanisms of atrophy and consequential regression of RV mass. Myostatin is a negative regulator of myocyte growth and likely mediates signaling at least in part via Erk1/2 (Yang et al., 2006). Although myostatin expression decreased in the male RV during HH, it did not normalize with reverse remodeling. Microarray gene expression analyses in RV from young animals that underwent PAB band regression identified growth factors, immune modulators, and apoptosis mediators as major cellular components underlying RV recovery (Boehm et al., 2020). Investigation of these mechanisms may help elucidate the mechanisms by which the removal of pathological load attenuates RV myocyte size with aging.

HH exposure stimulated RV fibrosis that was improved during normoxia re‐exposure. PH models such as chronic hypoxia exposure have been associated with deposition of collagen, the extent of which strongly correlates with RV dysfunction (Freed et al., 2012). The presence of biomarkers of collagen degradation is also associated with PH disease severity (Safdar et al., 2014). We show increased collagen deposition in response to HH exposure that is reversed in WK3RR and WK6RR for males and females, respectively. This finding is contrary to studies in young models of pressure overload where removal of the afterload stimulus (un‐banding the aorta) results in partial but not complete reversal of fibrosis (Neff et al., 2022). In this work, the authors demonstrate the initial degradation of collagen fibrils as responsible for reducing the fibrotic fraction. While we did not measure the activity of enzymes that degrade collagens, we did report mild increases in MMP1 expression during normoxia re‐exposure. Male SPARC gene expression was robustly upregulated in response to HH and mildly normalized by WK6RR. Cross‐linking of collagen increases its tensile strength and renders it more resistant to degradation (van der Slot‐Verhoeven et al., 2005) resulting in a stiffer and poorer performing heart. Therefore, while we found a reduction in collagen content in the RV in normoxia, the collagen still present in the RV has likely been crosslinked. While understudied in the RV, collagen cross‐linking in the LV increases with age (Thomas et al., 1992) has been associated with adverse outcomes in LV pressure overload (Herum et al., 2015), suggesting that collagen quality may be more important than the quantity. In hypertensive patients with chronic heart failure, the degree of collagen cross‐linking, not the collagen content per se, was associated with elevated filling pressures (Lopez et al., 2012). While these questions linking collagen cross‐linking and functional outcomes have not yet been studied in the aged or young RV, our data suggest that the aged PH RV phenotype may impair the ability of the RV to restore collagen and its function following removal of pathological load. Future mechanistic studies to dissect collagen turnover and its post‐translational cross‐linking should shed light into the contributions of fibrosis in RV aging and disease.

Despite regression of hypertrophy and fibrosis upon removal of pathological load, reverse remodeling of the aged RV did not result in improved RV systolic function. RV FAC and SV remained low in aged mice upon return to normoxia. Although it is not fully clear why RV function did not improve, age‐associated changes to the cardiovascular system likely blunt the ability of the aged RV to fully repair (Abdellatif et al., 2023). In a model of LV stress, Yanez‐Bisbe et al. demonstrated age‐related resistance to reverse remodeling. Using a model of β‐adrenergic (isoproterenol) induced LV failure, withdrawal of the hypertrophic stimulus resulted in complete reversal of the heart failure phenotype in young mice. However, aged animals still had a low LV ejection fraction (Yanez‐Bisbe et al., 2021). While conducted in LV‐centric disease, these data emphasize how aging may dysregulate the ability of the heart to repair itself following removal of stress. Current clinical treatments for PH focus on aggressive pulmonary vasodilator therapy with the hopes of consequently promoting RV reverse remodeling (D'Alto et al., 2020). Indeed, robust hemodynamic improvements (reduction in afterload) are required for reverse remodeling of the RV (Vizza et al., 2022). Many reports in young animals and human patients are consistent with this notion, with alleviation of RV afterload improving RV performance (Frist et al., 1995; Reesink et al., 2007). However, other reports suggest that even after the resolution of afterload, RV function does not fully normalize, even in some young models (Bonnet et al., 2004). The resolution of afterload may be even more important in aged models, given that reduction of RV afterload with normoxia during aging is complicated by mPAP increasing linearly with age (Davidson Jr. & Fee, 1990). Although it is not clear to what degree mPAP increases over the course of 6 weeks, it is feasible that age‐associated natural increases in afterload might offset normoxia reductions and thus blunt potential repair mechanisms in the aged RV.

Although neither male nor female mice completely reverse remodeled, we do report some sexual dimorphic outcomes with respect to the timing of reverse remodeling, with male mice generally remodeling quicker and more completely than female mice. PH and RV dysfunction have well‐established sex differences in disease outcomes, with PH patient registries consistently demonstrating better RV outcomes in young women than in men (Benza et al., 2010). Lower mortality in females has been explained by superior RV function in young women compared to men (Tandri et al., 2006; Tello et al., 2020). The importance of sex hormones in the RV is also widely reported in pre‐clinical models including Sugen hypoxia male rats that have worse RV function than females (Rafikova et al., 2015) and in monocrotaline‐induced PH where ovariectomized female rats experience less severe RV remodeling compared to males and control females (Nadadur et al., 2012). However, little is known regarding the interaction between age, sex, and RV function. Our previous work (McNair et al., 2023) and the present demonstrate similar hypertrophic, fibrotic, and functional outcomes in aged male and female mice. However, we did find that while both male and female mice underwent regressions in RV structure and function, the speed of the reverse remodeling was faster in aged male mice. The male RV showed lower collagen content at WK3 while this was not evident until WK6 in the female. The inability for the female heart to recover has also been reported in LV reverse remodeling studies. Administration of dual isoproterenol and angiotensin II stimulates LV hypertrophy. When these stimuli were subsequently removed, adult male mice saw rapid regression of LV hypertrophy, whereas females did not (Muehleman et al., 2022). Although studies investigating sex differences in RV reverse remodeling have not been explicitly performed, exogenous estrogen supplementation in ovariectomized mice demonstrates the potential beneficial effects of estrogen on the RV during PH recovery (Nadadur et al., 2012). While we did not measure estrogen in our animals, previous work in this aged cohort demonstrates significantly lower circulating estrogen (Yusifov et al., 2021). Together, it is possible that the loss of estrogen does not impact the response of the RV to HH, but it does limit the ability to reverse remodel. Given the growing number of older adults being diagnosed with PH (Hoeper et al., 2013), future mechanistic studies of the aging female RV should consider these sex differences.

## 5 LIMITATIONS AND CONCLUSIONS

It is difficult to account for the changes in chronological aging over the course of our experimental timeline. Mice in the present work aged 7–10 weeks, including the 4‐week HH exposure and the subsequent normoxia re‐exposure of 3–6 weeks. In the lifespan of a mouse, it is possible that this timeline may be sufficient to induce changes in the RV that are due to age, rather than hypoxia or normoxia per se. Although calculation of mouse‐human years is imperfect, 19–24 month‐old mice translate to 65–75 years old in humans, meaning that the mice in our cohort aged the equivalent of ~5 human years. We do note, however, that some of these limitations likely exist clinically as well. Long‐term interventions in human patients with PH and RV dysfunction will also have to overcome age‐related changes to RV structure and function to reverse remodel. Studies of reverse remodeling in the RV are sparse, and the field lacks a robust definition of what functional changes must occur to meet this definition. Most proposals include multiple variables chosen based on prognostic relevance, including RV FAC, RV EDV as well as right atrial area (Vizza et al., 2022). A recent suggestion defined RV reverse remodeling by improved RV EF accompanied by improvements in RV EDV and ESV (Hemnes et al., 2024). Given that RV EDV increases with advanced age (McNair et al., 2024), it will likely be difficult to parse apart changes with reverse remodeling on top of change associated with aging. Mechanistic studies to understand RV aging will aid in understanding how age impacts RV disease, recovery, and the identification of therapies for this unmet need.

In conclusion, we aimed to determine if the aged RV can reverse remodel following resolution of PH. We demonstrate that although the aged RV is capable of regressing RV myocyte size and fibrosis, structural and molecular adaptations are not sufficient to rescue PH‐induced declines in RV systolic function. This finding is in contrast to the adult RV that is capable of complete reverse remodeling with functional improvements, suggesting mechanisms of aging in the RV render it both more susceptible to severe RV disease (McNair et al., 2023) and unable to respond to potential therapeutic intervention. Future elucidation of the mechanisms of reverse remodeling is needed to inform age‐related RV‐directed therapies and to improve RV function in older populations.

## AUTHOR CONTRIBUTIONS

**Benjamin D. McNair**: Conceptualization, methodology, investigation, data curation, writing–original draft, writing—review and editing, funding acquisition. **Sushumna B. Satyanarayana**: Investigation, data curation, writing‐original draft, writing—review and editing. **Julian M. Matthews**: Conceptualization, investigation, data curation, writing—review and editing, funding acquisition. **Sydney M. Polson**: Data curation, formal analysis, writing—review and editing. **Emma R. Mehl**: Data curation, formal analysis. **Joshua P. Thornburg**: Data curation, formal analysis, writing—review and editing, **Danielle R. Bruns**: Conceptualization, methodology, investigation, data curation, writing—original draft, writing—review and editing, visualization, supervision, project administration, funding acquisition.

## FUNDING INFORMATION

This work was supported by NIA K01 AG058810 (DRB), American Heart Association 897622 (BDM), Rocky Mountain American College of Sports Medicine Student Grant (JMM), and Wyoming INBRE 2P20GM103432.

## CONFLICT OF INTEREST STATEMENT

The authors declare no conflicts of interest.

## ETHICS STATEMENT

All animal work was approved by the IACUC at the University of Wyoming.

## Supporting information


Figure S1.
