# Supplementary material for: Incomplete reverse remodeling in pulmonary hypertension‐induced right ventricular dysfunction in aged mice
Source: Physiol Rep. 2025 Jun 11;13(11):e70422. doi: 10.14814/phy2.70422 (PMC12159252; doi:10.14814/phy2.70422)
Supplement: Supplementary file 1 — Figure S1. [file PHY2-13-e70422-s001.pdf]

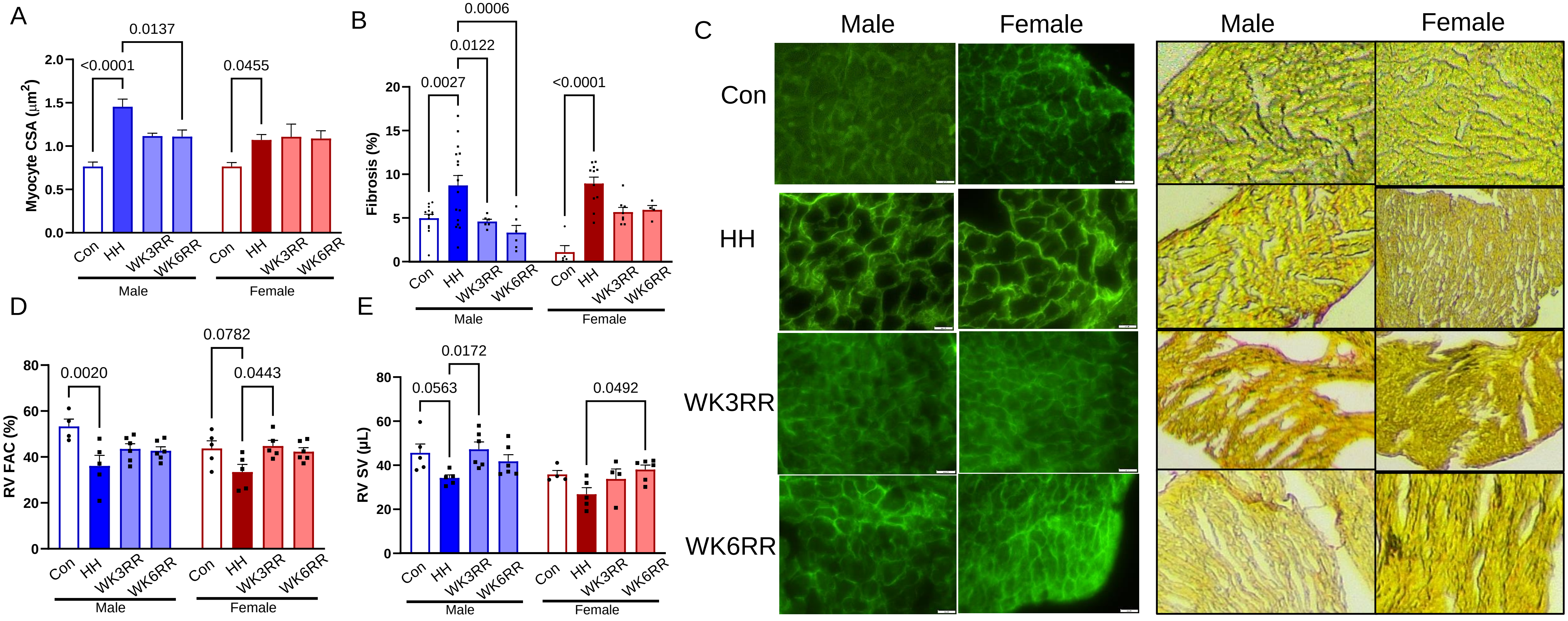

Supplementary Figure. Adult RV structural and functional remodeling in response to hypobaric hypoxia and reverse remodeling (RR) upon return to normoxia A) Myocyte cross-sectional area a RV cardiomyocyte cross sectional area (CSA) was higher in response to HH and reduced during normoxia exposure in males, during WK3RR, but did not regress in females. B) Collagen deposition increased upon hypoxic exposure and decreased by WK3RR in both sexes. C) Representative images of lectin staining and picro-Sirius red staining to show fibrosis. D) RV function by echocardiography demonstrated low RV Fractional Area Change (FAC) with HH that reversed in females with return to normoxia. E) RV stroke volume (RV SV) was lower in HH and recovered during WK3RR in males. Data were assessed by 2-way ANOVA. Data are expressed as mean ± SD. Adult mice were ~4 months of age at beginning of HH exposure.
